# The effectiveness of atorvastatin for the prevention of deep vein thrombosis in cancer patients undergoing chemotherapy: A randomised controlled trial: open label

**DOI:** 10.1186/s12959-023-00497-0

**Published:** 2023-05-08

**Authors:** Budi Setiawan, Widi Budianto, Tri Wahyu Sukarnowati, Daniel Rizky, Eko Adhi Pangarsa, Damai Santosa, Aru Wisaksono Sudoyo, Tri Indah Winarni, Ignatius Riwanto, Rahajuningsih Dharma Setiabudy, Catharina Suharti

**Affiliations:** 1 Hematology-Medical Oncology Division, Department of Internal Medicine, Faculty of Medicine, Universitas Diponegoro/Dr. Kariadi Hospital, Semarang, Indonesia; 2 Hematology-Medical Oncology Division, Department of Internal Medicine, Faculty of Medicine, Universitas Indonesia/Dr. Cipto Mangunkusumo Hospital, Jakarta, Indonesia; 3 Department of Anatomy, Faculty of Medicine, Universitas Diponegoro/Center for Biomedical Research (CEBIOR), Faculty of Medicine, Universitas Diponegoro, Semarang, Indonesia; 4 Digestive Surgery Division, Department of Surgery, Faculty of Medicine, Universitas Diponegoro/Dr. Kariadi Hospital, Semarang, Indonesia; 5 Department of Clinical Pathology, Faculty of Medicine, Universitas Indonesia, Jakarta, Indonesia

**Keywords:** Cancer, Chemotherapy, DVT, Thromboprophylaxis, Atorvastatin

## Abstract

**Background:**

Deep vein thrombosis (DVT) is a common complication in cancer. Although thromboprophylaxis in cancer patients is recommended by the guidelines, clinicians’ use of thromboprophylaxis remains limited due to cost, bleeding complications, and reluctance to give injectable anticoagulants. Inflammation plays essential roles in the pathogenesis of cancer-associated thrombosis. Owing to its ability to decrease proinflammatory cytokines, statins have anti-inflammatory properties. Thus, statins can be possibly utilized as thromboprophylaxis therapy in cancer patients undergoing chemotherapy.

**Objective:**

To compare the effectiveness of atorvastatin and rivaroxaban for DVT prevention in high-risk thrombosis patients with cancer undergoing chemotherapy.

**Methods:**

Double-blind, randomized controlled trial involving cancer patients with high-risk of thrombosis undergoing chemotherapy. We randomly assigned patients without deep-vein thrombosis at screening to receive atorvastatin 20 mg or rivaroxaban 10 mg daily for up to 90 days. Doppler ultrasonography was performed 90 days following chemotherapy to diagnose DVT. Average cost-effectiveness analysis was performed to analyze the cost of atorvastatin compared to rivaroxaban.

**Results:**

Of the eighty six patients who underwent randomization, primary efficacy end point was observed in 1 of 42 patients (2.3%) in the atorvastatin group and in 1 of 44 (2.2%) in the rivaroxaban group (Odds Ratio [OR], 0.953; 95% confidence interval [CI], 0.240 to 3.971; *p* = 1.000). There was a significant difference in the incidence of major bleeding, 2 of 42 patients (4.8%) in the atorvastatin group and 12 of 44 (27.3%) in the rivaroxaban group (OR, 0.257; 95% CI, 0.07 to 0.94; *p* = 0.007). The average cost-effectiveness ratio of using atorvastatin was lower than that of rivaroxaban.

**Conclusion:**

Atorvastatin did not differ significantly from rivaroxaban in reducing the incidence of DVT, lower bleeding risk, and cost-effectiveness for thromboprophylaxis in high-risk thrombosis patients with cancer undergoing chemotherapy. The presence of limited statistical power and wide confidence intervals in this study needs further study to strengthen the efficacy of atorvastatin as DVT prophylaxis in cancer patients.

**Trial registration:**

ISRCTN71891829, Registration Date: 17/12/2020.

**Supplementary Information:**

The online version contains supplementary material available at 10.1186/s12959-023-00497-0.

## Introduction

The incidence of Venous Thromboembolism (VTE) in cancer patients is high, regardless of whether they are receiving chemotherapy or not [1–5]. The majority of VTE events occurred immediately following chemotherapy initiation, 18.1% in the first month, 47% in the first 3 months, and 72.5% in the first 6 months [6]. Venous thromboembolism is a major cause of death, morbidity, delays in treatment, and increased costs of care [7]. The risk of death is also approximately threefold higher in asymptomatic DVT [8, 9].

Clinical studies have demonstrated the benefits and safety of VTE prophylaxis for patients; thus, supporting the current evidence-based recommendations for thromboprophylaxis in clinical practice [10–13]. Prophylaxis using VTE in cancer patients has been recommended by various guidelines [14–17]. However, in clinical practice, the use of VTE prophylaxis by clinicians to date remains very limited [18–21]. The most common reasons for this include cost considerations [18, 20, 22], fears of bleeding complications [19–21], other reasons due to lack of knowledge or confidence in thromboprophylaxis guidelines [19], lack of vigilance [20, 23], and reluctance to give daily injections of prophylactic anticoagulant [19].

The immune system and inflammation play an important role in the pathogenesis of cancer-associated VTE [24]. Cancer and chemotherapy administration can induce inflammatory conditions [25], which trigger the NF-κB signaling pathway to produce pro-inflammatory cytokines [26]. The role of pro-inflammatory cytokines such as CRP and IL-6 promotes the status of the immune system. Procoagulant works mainly by inducing TF expression [27], which triggers the coagulation system. This is characterized by increased levels of circulating thrombin and fibrin formation biomarkers such as F1+2 and D-Dimer [28, 29].

The anti-inflammatory properties of statins is generated by reducing proinflammatory cytokines and chemokines, highlighting its potential use as anti-thrombotic therapy [30] with a lower risk of bleeding compared to anticoagulants [31]. They are also cheaper and easier to administer.

Research data on statins and VTE in cancer patients are limited [32]. Previous studies in a prospective cohort demonstrated that statin administration and the incidence of VTE were low in patients with cancer. The role of statins in preventing VTE in cancer patients requires further confirmation of RCT studies [32]. Newman *et al*. reported data from 44 studies using oral atorvastatin in 16,495 patients. Severe side effects are rare and there have been no deaths with atorvastatin treatment [33].

Rivaroxaban is a factor Xa (FXa) inhibitor that inhibits FXa and prothrombinase activities, *thereby*effectively inhibiting thrombin formation. It not only functions in the coagulation cascade but also activates intracellular signaling pathways via G-protein-coupled PARs. Factor Xa primarily plays a role via PAR-2 and subsequently stimulates several intracellular NF-κB and MAPK signaling pathways that induce inflammation and fibrotic responses [34–37].

Rivaroxaban is an anticoagulant that is convenient to administer, with once a day dosing, while UFH (unfractionated heparin) and LMWH (low molecular weight heparin) are *injectables*, leading to higher compliance with rivaroxaban [38, 39]. Rivaroxaban has also been recommended for VTE prophylaxis in cancer patients by international and national guidelines [14–17]. The CASSINI study showed that thromboprophylaxis with rivaroxaban during the intervention period led to a lower incidence of thrombosis and lower bleeding side effects than placebo [13]. Moreover, it also does not require close monitoring during therapy [40, 41].

This study aimed to compare the effectiveness of atorvastatin and rivaroxaban for DVT prevention in high-risk thrombosis patients with cancer undergoing chemotherapy.

## Methods

### Patients

The eligibility criteria were cancer patients with a definite diagnosis of cancer based on histopathological examination, cancer patients who have not received any chemotherapy, high risk thrombosis patients (Khorana risk score ≥2), age 18-60 years old, and willing to participate in the study by signing the informed consent.

Exclusion criteria were deep vein thrombosis diagnosed with Doppler ultrasonography examination at baseline, within 14 days post-surgery, pregnancy, taking an anti-thrombotic drug, congenital conditions altering the coagulation system, creatinine clearance <30 ml/minute, patients with AST level >3 times the upper normal limit, patients with total bilirubin of >5 mg/dl, patients with CK >3 times the upper normal limit, performance status ECOC of ≥3, patients with cardio-cerebrovascular disease, patients with infection, patients with active, major, serious, life-threatening bleeding that cannot be overcome with medical or surgical intervention, especially in a critical area (intra-cranial, pericardial, retroperitoneal, intra-ocular, intra-articular, intraspinal), malignant hypertension, congenital coagulopathy or severe platelet dysfunction, severe and persistent thrombocytopenia (<20,000/μl).

### Trial design and interventions

This double-blind, randomized controlled trial involving high-risk thrombosis patients with cancer undergoing chemotherapy is conducted in Dr. Kariadi hospital, the main teaching hospital for Faculty of Medicine of Diponegoro University, Semarang, Indonesia. The hospital is a tertiary referral hospital for all patients with cancer in Central Java province.

The sample size was calculated with two sampel proportion test formula with α: 5%, β: 20%. Since no previous research has been conducted on this matter, the researcher cannot determine a reference for the difference in proportion as one of the components for calculating the sample size. Based on the researcher’s judgement, the difference in proportion is 26%. After calculating the risk of drop out of 10% each group, the required samples for each group was 40.

Patients meeting the inclusion criteria were selected as the subjects of this study. Before this study was conducted, all patients had been informed about the study in details during individual interviews and asked to sign a written informed consent. The patient’s history, especially cancer history, tumor site, tumor histology and tumor stage were subsequently documented. Age, gender, ABO blood group, body mass index, ECOG, Khorana score, chemotherapy regimen were also recorded carefully.

The subjects were randomized into 2 groups. After receiving a prescription with a study code from the investigator, the patient will go to the pharmacy and received a 30 days drugs without packaging. The study employed a simple random sampling method using a list of random numbers generated based on sample size. Once recruited, patients will be asked to select an envelope from a basket, which will indicate their allocation to either the control or intervention group using a 1:1 randomization ratio. The study utilized third-party randomization conducted by the pharmacist. This involved concealing and randomly allocating the study drugs to participants. The pharmacist then distributed the drugs to the participants after removing them from their original packaging and placing them in identical containers for both groups. This should be done to minimize potential bias resulting from the different shapes of atorvastatin and rivaroxaban tablets. Stratification of the sample was not performed in this study. The investigator was allowed to open the concealment, whenever a serious adverse event occurred. The intervention group receiving chemotherapy and atorvastatin tablets 20 mg/24 hours for up to 3 months during chemotherapy. The control group received chemotherapy and rivaroxaban 10 mg/24 hours for up to 3 months during chemotherapy. On the 7th day, a physical examination was performed to see signs of impaired liver function and signs of myopathy. Liver function laboratory tests (ALT, AST) were performed to look for signs of impaired liver function. CK examination is done if there are signs of myopathy. If there is an increase in AST levels, ALT 3 times the upper limit and CK levels 3 times the upper limit, the study treatment would be stopped. If no side effects of drug administration were observed on the 7^th^ day, further laboratory tests are performed once a month.

Monitoring of signs of DVT was performed by calculating the Wells’ score on days 30 and 60, and patients with Wells’ score of 2 underwent further Doppler ultrasound examination. On day 90, all patients underwent Doppler ultrasound to assess the incidence of DVT. Doppler ultrasound examination was performed within ±7 days of the specified time. During those period, we also monitored for signs of bleeding. The results are recorded on a pre-established research form. After the pre-set number of research subjects is reached or the end of the research deadline is completed, data is aggregated, and statistical analysis are conducted.

This study was approved by Dr. Kariadi Hospital Institutional Review Board, as stated in the Ethical Clearance Statement number 665/EC/KEPK-RSDK/2020. The study registry number is ISRCTN71891829, Registration Date : 17/12/2020.

### Prediction score

Two prediction scores were used in this study: the Khorana and the Wells’ score. For each patients, we calculated the Khorana risk score to stratify the risk of VTE in cancer patients undergoing chemotherapy [42]. Patients were assigned to three risk categories for VTE: low risk (score 0), intermediate risk (scores 1-2), and high-risk (score ≥ 3). In this study, cancer patients are deemed to have high risk of thrombosis if the patient had a Khorana score of ≥ 2 [14].

Wells’ score considers 1 point each for active cancer, paralysis, paresis, recent plaster immobilization of lower limb, recently bedridden for > 3 days, major surgery in the past 4 weeks; localized tenderness along the distribution of deep venous system; entire leg swollen; calf swelling >3 cm compared to asymptomatic leg; pitting edema and collateral superficial veins. The score is subtracted by 2 points for alternative diagnosis as likely as, or more likely than DVT. A score of 3 or higher suggest that DVT is likely and patients should receive a diagnostic ultrasound and the result will be documented [43].

### Outcome measures

The primary efficacy endpoint was the occurrence of proximal or asymptomatic DVT of the limbs diagnosed objectively through Doppler ultrasound of the lower limbs; symptomatic DVT of the upper extremities or distal DVT of the lower limbs; symptomatic or incidental pulmonary embolism; and death from VTE. The primary efficacy endpoint of the study was the occurrence of DVT confirmed by duplex ultrasound.

The secondary efficacy endpoint is the occurrence of symptomatic VTE events and clinically relevant conditions that are not included in the primary efficacy endpoint, such as death from any causes, observed arterial thromboembolism, and observed visceral thromboembolism.

Color duplex sonography was performed at the Department of Radiology of Dr. Kariadi Hospital, Semarang, Indonesia. Patients with clinically suspected DVT and Wells’ score of ≥ 2 was assessed for DVT using Loqic 7 pro US imaging system (Loqic 7 pro; GE Healthcare, USA) with the 7-10 Hz linear probe. The diagnosis of DVT was based either on presence of a non-compressible segment (compression ultrasound test – CUS) or flow impairment on color Doppler imaging. Patients were examined for both proximal (popliteal, femoral, and common femoral vein) and distal (peroneal and tibial veins) DVT.

The primary safety endpoint is the occurrence of major bleeding that meets the criteria of the International Society on Thrombosis and Hemostasis (ISTH). Major bleeding was defined as clinically evident bleeding associated with one or more of the following [44]: 1) Fatal bleeding, and/or 2) Symptomatic bleeding at critical sites such as intracranial, intraspinal, intraocular, pericardial, intra-articular, intramuscular with compartment syndrome, retroperitoneal, intra-articular or pericardial, or intramuscular with compart-ment syndrome, and/or 3)Bleeding causing a decrease in hemoglobin of 2 g/dL or more, or, requires transfusion of 2 or more units of whole blood or red blood cells.

Secondary safety endpoints were the percentage of patients with clinically relevant non-major bleeding such as ISTH criteria, minor bleeding, and bleeding during the intervention period. Clinically relevant non-major bleeding was defined as actual bleeding that did not meet the criteria for major bleeding but was associated with [13]: 1) medical intervention, 2) unscheduled contact (visit or phone call) with a physician, 3) temporary discontinuation of the study drug, or, 4) discomfort such as pain, or interference with activities of daily living.

The hypotesis of this study was the efficacy of thromboprophylaxis in cancer patients at high risk of thrombosis undergoing chemotherapy with atorvastatin was comparable to rivaroxaban.

### Response to treatment and follow-up

All patients undergoing chemotherapy were followed up for 3 months. Patients were either evaluated during routine visits at the hematology and medical oncology outpatient clinic or medical ward at every pre- and post-chemotherapy cycle. Performance status, chemotherapy eligibility and Wells’ score are assessed at each visit. DVT occurring after enrollment was documented as a new event.

### Statistical analysis

Analysis of the effectiveness of atorvastatin compared to rivaroxaban as prophylaxis of deep vein thrombosis in cancer patients at high risk of thrombosis undergoing chemotherapy was performed using Intention-to-Treat Analysis (ITT) [45].

The data collected were processed and analyzed descriptively. Descriptive statistical analysis is used to describe or provide an overview of the study. In this descriptive analysis, variables data are presented in a table to test the equality of the mean values and the frequency distribution of the variable values ​​in the population [46]. An analysis to see the effect of atorvastatin compared to rivaroxaban on the incidence of DVT was performed using chi-square test. Data were analyzed and interpreted to test the proposed hypothesis using SPSS statistical software (IBM v. 21; SPSS Inc., USA). The *p* value of < 0.05 is considered statistically significant.

A cost-effectiveness analysis of the model was performed by comparing two drugs for thromboprophylaxis: (1) Atorvastatin 20 mg/24 hours, and (2) Rivaroxaban 10 mg/24 hours. The analysis was conducted from a healthcare system perspective, with the primary endpoint being cost per patient without DVT [47].

## Results

### Demographics and characteristics of the study population

From January 2021 to December 2021, 348 new cancer patients were screened for their clinical diagnosis, histopathological data, and whether they will undergo chemotherapy or not. There were 106 subjects who met the inclusion criteria. Of the 106 subjects, there were 86 who entered the intention to treat population and were randomized.

**Fig. 1:**
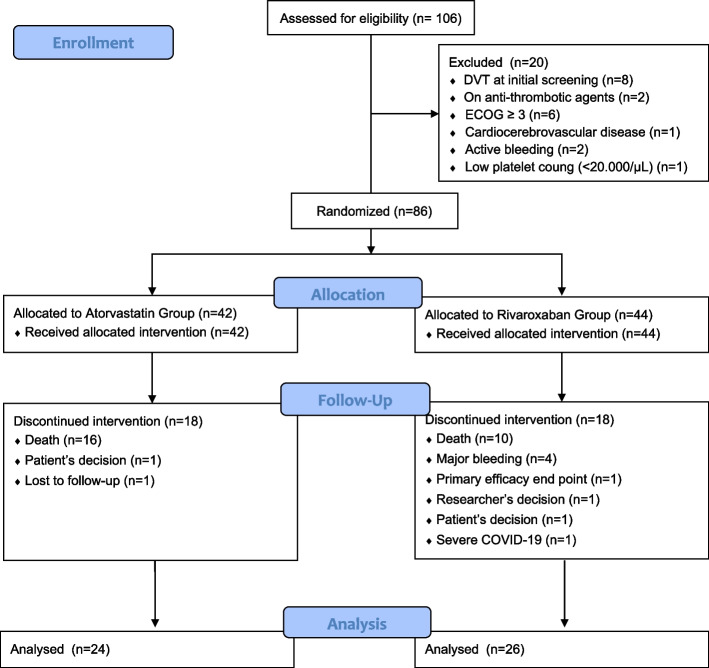
Study consort

In the treatment group, namely the group that received atorvastatin 20 mg daily, 18 subjects stopped receiving study treatment before the end of the study. In the control group, which received rivaroxaban 10 mg daily, 18 study subjects stopped receiving the study drug before the end of the study. The CONSORT flow diagram can be seen in Fig. 1.

Demographics and baseline characteristics of the study population are shown in Table 1. Of the 106 patients who met the inclusion criteria, 8 (6.7%) patients had DVT at the initial screening. There were no significant differences in median age and sex in the atorvastatin and rivaroxaban groups. There were no significant differences in blood group, body mass index, ECOG, Khorana score, cancer incidence, stage at diagnosis, chemotherapy regimen and laboratory parameters of hemoglobin, leukocytes, and platelets between the atorvastatin group and the rivaroxaban group.

**Table 1:** Characteristics of the trial population at baseline

Characteristcs	Atorvastatin *n* = 42	Rivaroxaban *n* = 44	Total *n* = 86	*p*
**Age (yr), median (min – max)**	43.5 (19–60)	41.5 (20–60)		0.118^*^
**Sex, no. (%)**				
Male	21 (50%)	24 (54.5%)	45	0.837^*^
Female	21 (50%)	20 (45.5%)	41	
**Blood type, no. (%)**				
O	19 (45.2%)	17 (38.6%)	36	0.688^*^
Non-O	23 (54.8%)	27 (61.4%)	50	
**Body Mass Index (kg/m**^**2**^**), no. (%)**				
Underweight	14 (33.3%)	20 (45.5%)	34	0.107^⁋^
Normoweight	21 (50%)	22 (50%)	43	
Overweight/obesity	7 (16.7%)	2 (4.5%)	9	
**ECOG, no. (%)**				
0	26 (61.9%)	29 (52.7%)	55	0.911^⁋^
1	12 (28.6%)	8 (18.2%)	20	
2	4 (9.5%)	7 (15.9%)	11	
**Khorana score, no. (%)**				
Intermediate risk (2)	23 (54.8%)	32 (72.7%)	55	0.131^*^
High risk (≥ 3)	19 (45.2%)	12 (27.3%)	31	
**Primary site of cancer, no. (%)**				
** Very high risk of thrombosis**				
Pancreas	5 (11.9%)	2 (4.5%)	7	0.109^⁋^
Stomach	2 (4.8%)	1 (2.3%)	3	
**High risk of thrombosis**				
Lung	8 (19%)	7 (15.9%)	15	
Genitourinary	3 (7.1%)	2 (4.5%)	5	
Gynecology	0 (0%)	2 (4.5%)	2	
Limphoma	4 (9.5%)	5 (11.4%)	9	
**Average risk of thrombosis**				
Colorectal	14 (33.3%)	17 (38.6%)	31	
Breast	2 (4.8%)	2 (4.5%)	4	
Sarcoma	0 (0%)	2 (4.5%)	2	
Others	4 (9.5%)	4 (9.1%)	8	
**Stage of cancer at diagnosis, no. (%)**				
I	2 (4.8%)	4 (9.1%)		0.962^⁋^
II	5 (11.9%)	5 (11.4%)		
III	13 (31%)	11 (25%)		
IV	22 (52.4%)	24 (54.5%)		
**Chemotherapy regimen, no. (%)**				
5FU *Based*	20 (47.6%)	20 (45.5%)		0.446^⁋^
Cisplatin *Based*	14 (33.3%)	14 (31.8%)		
R-CHOP	1 (2.4%)	4 (9.1%)		
BEP	1 (2.4%)	1 (2.3%)		
Taxane *Monotherapy*	2 (4.8%)	2 (4.6%)		
Anthracycline *Based*	1 (2.4%)	2 (4.6%)		
ABVD	1 (2.4%)	1 (2.3%)		
GRALL-LYSA	0 (0%)	1 (2.3%)		
De Angelis	0 (0%)	1 (2.3%)		
**Laboratory parameters**				
Haemoglobin (g/dL)	11.05 (7.7–15.3)	11.25 (7.3–15.9)		0.659^⁋^
Leukocyte (× 10^3^/uL)	13 (4.9–37)	11.9 (5.1–21.8)		0.169^⁋^
Platelet (× 10^3^/uL)	445 (194–707)	465 (238–951)		0.883^⁋^

^*^Chi Square Test

^⁋^Mann–Whitney U Test

Of the 86 subjects, in the atorvastatin group there were 18 (42.8%) study subjects who discontinued the study treatment, and in the rivaroxaban group there were 18 (40.9%) study subjects who discontinued the study. There was no significant difference between the number of atorvastatin and rivaroxaban groups who could not continue the study treatment (Odds Ratio [OR], 1.042; 95% confidence interval [CI], 0.674-1.611; *p* = 1.000) (Table S 1 in the Supplementary Appendix).

The study treatment was permanently discontinued early before the study was completed for several reasons. The reasons for discontinuing the study treatment include death in 16 (88.9%) and 10 (55.6%) study subjects in the atorvastatin and rivaroxaban groups, respectively; due to major bleeding in 0 (0%) and 4 (22.2%) subjects, respectively; due to primary efficacy endpoint in 0 (0%) and 1 (5.6%) subjects, respectively; due to patients’ decisions in 1 (5.6%) and 1 (5.6%) study subjects, respectively; due to loss to follow-up in 1 (5.6%) and 0 (0%) research subjects, respectively; due to the researcher's decision in 0 (0%) and 1 (5.6%) study subjects, respectively; and due to severe COVID-19 infection in 1 (5.6) %) and 0 (0%) subjects, respectively. A significant difference between the two groups was observed in terms of reasons for stopping the study treatment (*p* = 0.043). Patients who stopped receiving study treatment were followed up to see the primary efficacy endpoint for up to 90 days of observation period. Table of rates and reasons for early discontinuation before study completed in the atorvastatin and rivaroxaban groups can be seen in Table S 2 in the Supplementary Appendix.

One study subject in the rivaroxaban group who withdrew from the study eventually died before 90 days of observation is completed. There were 11 study subjects who died in the rivaroxaban group. The majority of death in the atorvastatin group occurred in less than 30 days of observation. This showed that death is not due to the study drug, because the duration of study drug consumption remains minimal. The main cause of death in both the atorvastatin and rivaroxaban groups was cancer progression. In the atorvastatin group, there was 1 patient who died of severe COVID-19 infection in the COVID-19 isolation ICU.

Demographics and baseline characteristics of the study population who discontinued the study can be seen in Table S 3 in the Supplementary Appendix. There was no significant difference between the atorvastatin group and the rivaroxaban group in terms of age, sex, blood type, body mass index, ECOG score, Khorana score, chemotherapy regimen, and laboratory parameters (*p* > 0.05).

In the study population who stopped receiving study treatment, there was a significant difference between the atorvastatin group and the rivaroxaban group in terms of primary site of cancer and the stage at diagnosis with *p* = 0.003 and *p* = 0.043, respectively. The primary location of most cancers in the atorvastatin group was the lung (*n*=5, 27.8%) , while in the rivaroxaban group were colorectal cancers (*n*=7, 38.9%). Based on the stage at diagnosis, most of the patients were at stage IV in both the atorvastatin and rivaroxaban group (*n*=8 (44.4%) and *n*=14 (77.8%), respectively), with the higher number being in the rivaroxaban group.

Demographics and baseline characteristics of the study population who continued the study are shown in Table S 4 in the Supplementary Appendix. There were no significant differences between the atorvastatin group and the rivaroxaban group in terms of age, sex, blood type, body mass index, ECOG score, primary location of cancer, stage at diagnosis, chemotherapy regimen, and laboratory parameters (*p* > 0.05).

There was a significant difference between the atorvastatin group and the rivaroxaban group in terms of Khorana score (*p* = 0.006). In the rivaroxaban group, 23 (88.5%) patients had Khorana score of 2 and in the atorvastatin group 13 (45.8%) patients had Khorana score of 3.

### The effects of atorvastatin on the incidence of deep vein thrombosis

#### Primary efficacy endpoint

The effect of atorvastatin administration was observed using intention-to-treat analysis over a 90-day observation period, regardless of whether the incidence of deep vein thrombosis occurred after discontinuation of the study drug. All study subjects who had discontinued the study drug before the end of the study underwent a Doppler ultrasound examination on the 90^th^ day.

**Fig. 2:**
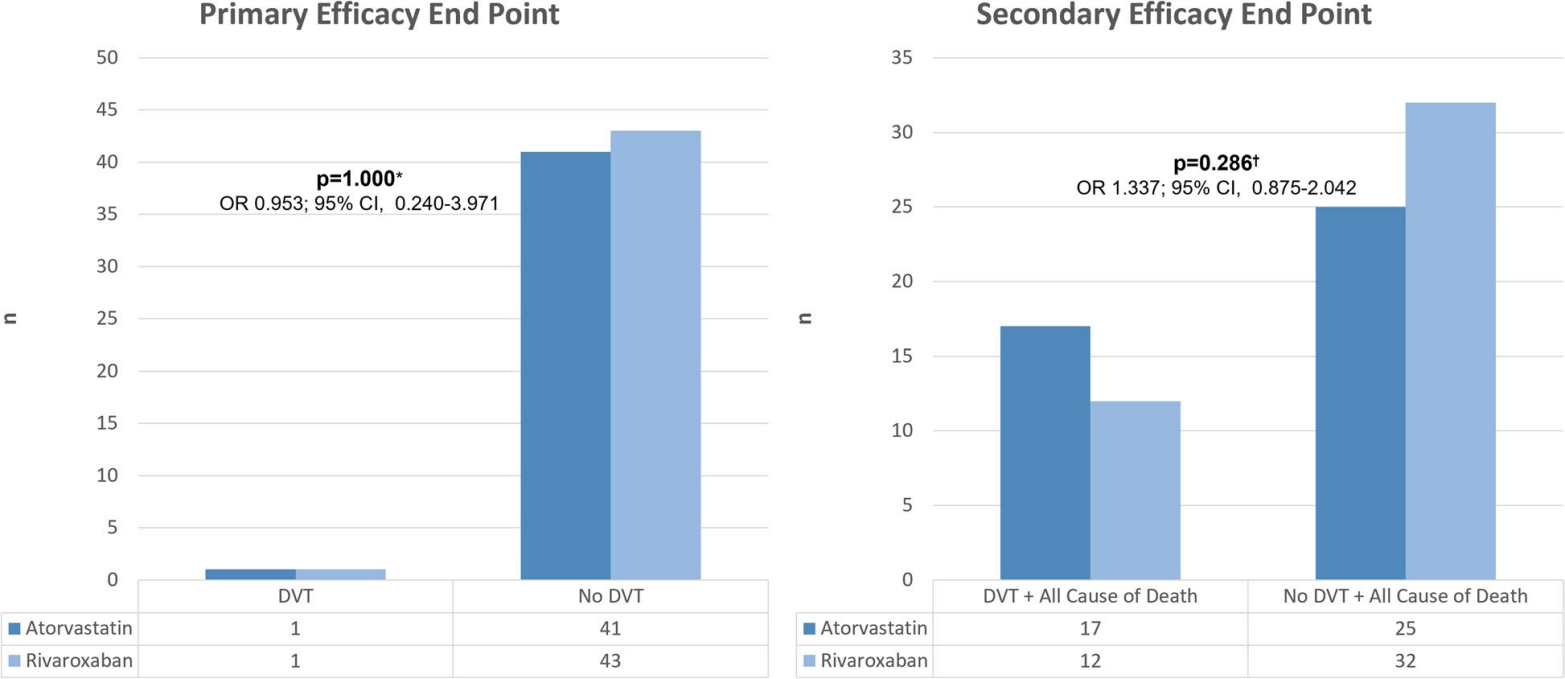
Primary and secondary efficacy end points. *Fisher’s Exact Test, †Chi Square Test

In this study, there was 1 (2.3%) and 1 (2.2%) DVT case in the atorvastatin and rivaroxaban group, respectively (OR 0.953; 95% CI, 0.240-3.971; *p* =1,000) (Table 2). The bar chart can be seen in Fig. 2.

**Table 2:** Study efficacy end points

Efficacy endpoints	Atorvastatin *N* = 42	Rivaroxaban *N* = 44	*p*	OR;95% CI
Primary efficacy end points				
Yes	1 (2.3%)	1 (2.2%)	1.000*	0.953; 0.240–3.971
No	41 (97.7%)	43 (97.8%)		
Secondary efficacy end points				
Yes	17 (40.5%)	12 (27.3%)	0.286^†^	1.337; 0.875–2.042
No	25 (59.5%)	32 (72.7%)		

^*^Fisher’s Exact Test

^†^Chi Square Test

#### Secondary efficacy endpoint

In this study, the secondary efficacy endpoint were as follows: 1 case of DVT and 16 deaths (17 [40.5%] cases) in the atorvastatin group, and 1 case of DVT and 11 deaths (12 [27.3%] cases) in the rivaroxaban group (OR 1.337; 95% CI, 0.875-2.042; *p* = 0.286) (Table 2). The bar chart can be seen in Fig. 2.

#### Primary safety endpoint

**Fig. 3:**
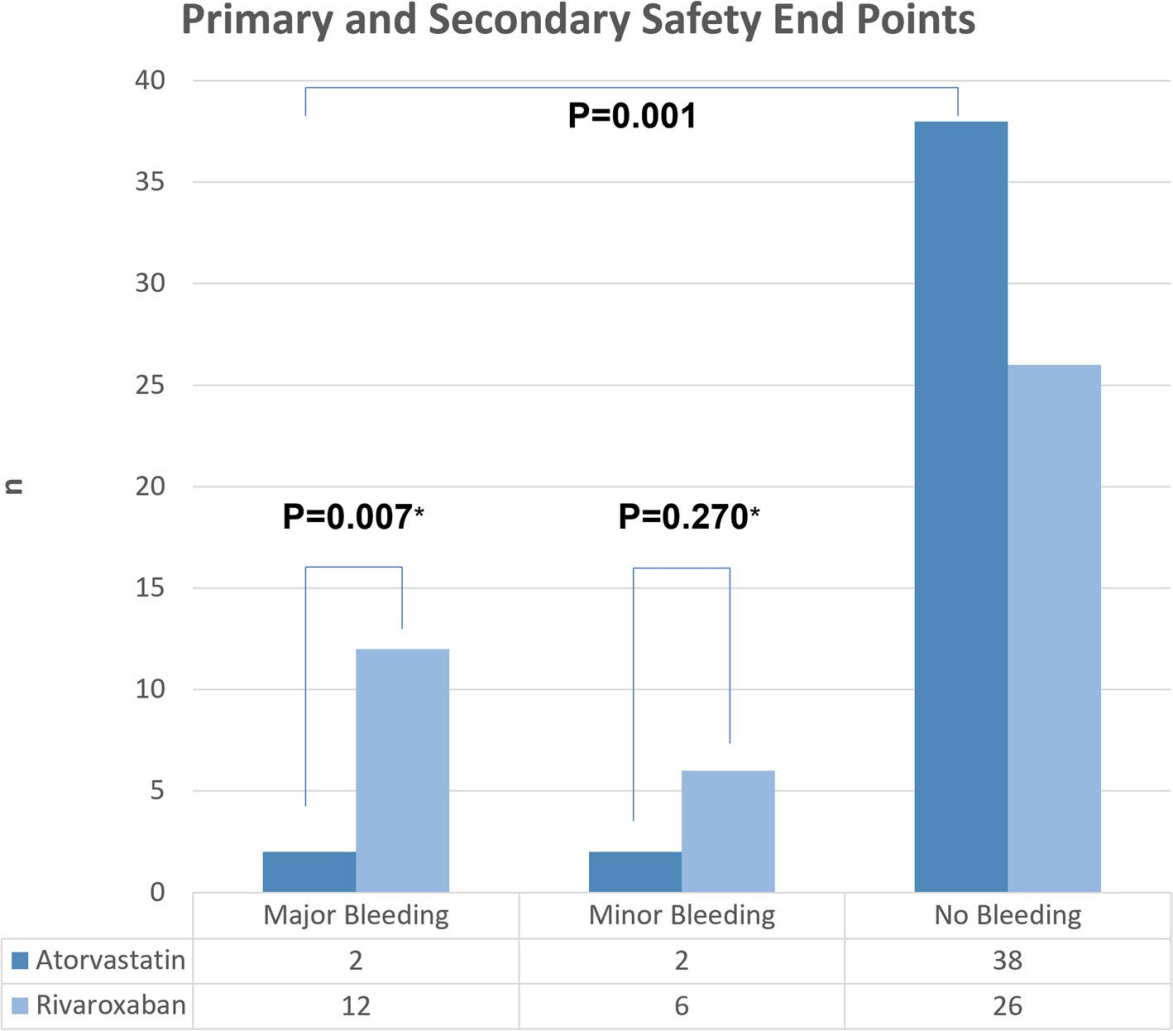
Primary and secondary safety end points. *Fisher’s Exact Test, ⁋Mann Whitney

In this study, the primary safety endpoint during the 90 day observation period occurred in 2 (4.8%) and 12 (27.3%) subjects in the atorvastatin and the rivaroxaban group, respectively. There was a significant difference in terms of major bleeding incidence between the atorvastatin group and the rivaroxaban group (OR 0.257; 95% CI, 0.07-0.94; *p* = 0.007) (Table 3 and Fig. 3).

**Table 3:** Study safety end points

Efficacy end points	Atorvastatin *N* = 42	Rivaroxaban *N* = 44	*p*	OR;95% CI	*p*
Primary safety end points					
Yes	2 (4.8%)	12 (27.3%)	0.007*	0.257; 0.070–0.940	
No	40 (95.2%)	32 (72.7%)			
Secondary safety end points					
Yes	2 (4.8%)	6 (13.6%)	0.270*	0.320; 0.600–1.670	0,001^*⁋*^
No	40 (95.2%)	38 (86.4%)			
No Bleeding	38 (90,5%)	26 (59,1%)			

^*^Fisher’s Exact Test

^⁋^Mann Whitney U Test

#### Secondary safety endpoints

In this study, the secondary safety endpoint during the 90 day observation period occurred in 2 patients (4.8%) and in 6 patients (13.6%) in the atorvastatin and the rivaroxaban group, respectively. There was no clinically-relevant significant difference in terms of non-major bleeding incidence between the atorvastatin and rivaroxaban groups (OR 0.32; 95% CI, 0.60-1.67; *p* = 0.27) (Table 3 and Fig. 3).

There was a significant difference in terms of major bleeding incidence and clinically-relevant non-major bleeding in the atorvastatin group and rivaroxaban groups, with *p* = 0.001 (Fig. 3).

Bleeding occurred in 22 (25.6%) patients. The locations of major and non-major bleeding in the atorvastatin and rivaroxaban groups can be seen in Table S 5 in the Supplementary Appendix. The major bleeding events occurred in 12 patients in the rivaroxaban group characterized by clinically significant bleeding in gastrointestinal tract or cancer primary site, and a decrease in hemoglobin levels of 2 g / dL or more requiring transfusion of 2 or more units of blood cells (ISTH criteria). The majority of clinically-relevant non-major bleeding occurred in 5 (22.7%) patients in the rivaroxaban group, the most being in the lower gastrointestinal tract.

### Evaluation of side effects of atorvastatin

During the 90-day observation period, an evaluation of the presence of liver function toxicity and signs of myopathy was performed. There were no signs of liver toxicity and myopathy progressing to fatal or nonfatal rhabdomyolysis during the 90-day observation period. Mann-Whitney U Test showed no significant difference in ALT and AST levels between the atorvastatin group and the rivaroxaban group until 90 days of observation (*p* = 0.565 and *p* = 0.156, respectively). The Mann-Whitney U Test showed no significant difference in total bilirubin levels between the atorvastatin group and the rivaroxaban group until 90 days of observation (*p*=0.245) (Table 4).

**Table 4:** Baseline data and 90^th^ day of liver function levels, blood lipid profile and myopathy

	**Variable**	**Atorvastatin *n* = 42** ***Mean* ± SD**	**Rivaroxaban *n* = 44** ***Mean* ± SD**	***p***
**Pre-treatment**	SGOT(mg/dL)	35,26 ± 21,44	36,98 ± 25,60	0,829^†^
	SGPT (mg/dL)	29,93 ± 40,32	28,55 ± 30,04	0,772^†^
	Total Bilirubin (mg/dL)	0,78 ± 0,87	0,69 ± 0,65	0,872^†^
	Triglycerides(mg/dL)	158,74 ± 112,07	163,09 ± 78,66	0,265^†^
	Total cholesterol (mg/dL)	176,17 ± 57,70	181,09 ± 72,87	0,890^†^
	LDL cholesterol (mg/dL)	132,45 ± 42,08	131,66 ± 68,92	0,455^†^
	HDL cholesterol (mg/dL)	36,20 ± 10,95	36,91 ± 17,48	0,424^†^
**90**^**th**^** day**	Signs of myopathy	0	0	-
	SGOT(mg/dL)	50,69 ± 53,58	44,43 ± 38,90	0,565^†^
	SGPT (mg/dL)	45,52 ± 62,97	29,09 ± 24,10	0,156^†^
	Total Bilirubin (mg/dL)	0,80 ± 0,73	0,94 ± 1,69	0,245^†^
	Triglycerides (mg/dL)	148,45 ± 92,92	175,93 ± 96,69	0,095^†^
	Total cholesterol (mg/dL)	149,76 ± 52,61	187,89 ± 50,38	0,000^†^
	LDL cholesterol (mg/dL)	91,48 ± 39,57	132,07 ± 49,25	0,000^‡^
	HDL cholesterol (mg/dL)	34,60 ± 14,43	40,68 ± 30,97	0,385^†^

^†^Mann–Whitney U Test

^‡^Independent T Test

The Mann-Whitney U Test showed that there was no significant difference in triglyceride levels between the atorvastatin group and the rivaroxaban group until 90 days of observation (*p*=0.095). The test also showed that there was no significant difference in HDL cholesterol levels between the atorvastatin group and the rivaroxaban group until 90 days of observation (*p*=0.385), while there was a significant difference in total cholesterol levels between the atorvastatin and rivaroxaban groups up to 90 days of observation (*p*=0.000). Independent T-Test showed that there was a significant difference in LDL cholesterol levels between the atorvastatin group and the rivaroxaban group until 90 days of observation (*p* = 0.000). Total cholesterol and LDL cholesterol levels up to 90 days of observation were lower in the atorvastatin group compared to the rivaroxaban group, but the average total cholesterol and LDL cholesterol levels were still within the normal range of 149.76 mg/dL (normal value: < 200 mg/dL) [48]. and 91.48 mg/dL (normal value: < 100 mg/dL) [49, 50] (Table 4).

### Cost-effectiveness analysis

This study showed that administering 20 mg atorvastatin daily was cheaper compared to 10 mg rivaroxaban daily for 3 months as thromboprophylaxis of DVT events in cancer patients at high risk of thrombosis undergoing chemotherapy.

The atorvastatin group in this study used Lipitor® 20 mg (atorvastatin 20 mg) and Xarelto® 10 mg (Rivaroxaban 10 mg) daily for a 90 day observation period. On day 90, one of the 42 patients in the atorvastatin group was discovered to have DVT during the Doppler ultrasound evaluation, resulting in 41 subjects without DVT events during the 90-day observation period.

In the rivaroxaban group there were 44 patients who took the drug for up to 90 days of observation period. There was an incidence of DVT in the rivaroxaban group on the 27^th^ day of observation. The study drug with a prophylactic dose of thrombosis was then changed to a therapeutic dose, so that in the rivaroxaban group there were 43 study subjects without DVT events.

In addition to using atorvastatin and rivaroxaban anticoagulants, the study of thrombosis prophylaxis in cancer patients also requires other therapy, including intravenous fluids, antiallergic drugs, antiemetic drugs and chemotherapy regimens. Due to the variability of the cancer, different chemotherapy regimens were given to the patients, with differing costs. Thus, to calculate the cost-effectiveness of treatment in this study, only atorvastatin and rivaroxaban prices were used to calculate the cost of treatment.

Several stages are carried out in the cost-effectiveness analysis, with the first step being the comparison of the average total cost with effectiveness. The average total cost was calculated from the cost of atorvastatin and rivaroxaban. The average total cost of thrombosis prophylactic therapy in cancer patients using atorvastatin was IDR 2,016,000.00 and the average total cost of prophylactic therapy in cancer patients using rivaroxaban was IDR 4,050,000.00. The effectiveness of the study treatment can be seen in Table 5, with the therapeutic effectiveness of atorvastatin being 97.6% while the effectiveness of rivaroxaban being 97.7%.

**Table 5:** Cost effectiveness analysis

Group	Total Cost for 90 days (IDR)	Therapy Effectiveness	ACER (IDR)
**Atorvastatin**	IDR 2,016,000	97.6%	IDR 20,655
**Rivaroxaban**	IDR 4,050,000	97.7%	IDR 41,453

The second step of the CEA is to calculate the Average Cost-Effectiveness Ratio (ACER) of each group. Calculations were performed using the ACER formula (average total cost of therapy divided by effectiveness), resulting the ACER value in cancer patients who used atorvastatin being IDR 20,655.00 and in patients taking rivaroxaban being IDR 41,453.00 (illustrated in Table 5).

The third stage of this analysis is the positioning of alternative thrombosis prophylaxis based on the cost-effectiveness diagram (Figure S 1 in the Supplementary Appendix) [47]. The positioning of alternative treatments is seen from the average total cost of therapy and effectiveness. The desired alternative for thrombosis prophylaxis in cancer patients in this study is atorvastatin. the position of atorvastatin is in column D, which means that atorvastatin has similar effectiveness and lower cost compared to rivaroxaban, so there is no need to calculate Incremental Cost-Effectiveness Ratio (ICER).

The analysis showed that atorvastatin was more cost-effective than rivaroxaban as seen by the ACER (IDR 20,655.00 per effectiveness vs. IDR 41,453.00 per effectiveness).

## Discussion

Of the 86 study subjects, there were 18 (42.8%) patients in the atorvastatin group who discontinued the study, and there were 18 (40.9%) patients in the rivaroxaban group who discontinued the study. There was no significant difference between the number of subjects who discontinued the study in the atorvastatin group and the rivaroxaban group (OR 1.042; 95% CI 0.74-1.611; *p*=1.000). This showed that the treatment group and the control group were similar in terms of the number of subjects who stopped participating the study.

The main cause of death in both the atorvastatin and rivaroxaban groups was the progression of cancer. In the atorvastatin group, there was 1 patient who died of severe COVID-19 infection in the COVID-19 isolation ICU. In the rivaroxaban group, the main reason of drug discontinuation was major bleeding, which, if not stopped, would have been harmful for the study subjects. This indicates that the risk of bleeding was higher in the rivaroxaban group than in the atorvastatin group.

Early discontinuation of the study drug occurred in 42% of study subjects. This is due to the fact that most research subjects were already in advanced stage of cancer. This finding is consistent with other large-scale studies investigating thrombosis prophylaxis in cancer patients, such as the CASSINI [13], PROTECHT [51] and SAVE-ONCO [52] studies. In the CASSINI study, discontinuation of the study drug before the end of the study occurred in 47% of the subjects, higher than in this study [13].

In the study population who discontinued the study, there was a significant difference between the atorvastatin group and the rivaroxaban group in terms of the primary site of cancer and the stage at diagnosis (*p*=0.003 and *p*=0.043, respectively). The most common primary location of cancer in the atorvastatin group was the lung (27.8%) while in the rivaroxaban group, 38.9% patients had colorectal cancer. Based on the stage at diagnosis, the most common stage at diagnosis was stage IV (44.4% and 77.8% in the atorvastatin and rivaroxaban group, respectively), with the higher percentage being in the rivaroxaban group.

The data above shows that, from the study population who discontinued the study treatment, the most frequent cancer was lung and colorectal cancers and the most frequent stage at diagnosis was stage IV. This is consistent with data from a UK cancer study , which showed that the 1-year survival rate for lung cancer was highest at stage I and the lowest at stage IV at 88% and 19%, respectively [53]. Data from ASCO’s Cancer.net showed that if colorectal cancer was diagnosed in stage I, the survival rate was 90%. If colorectal cancer has spread to surrounding tissues or organs and/or regional lymph nodes (stage II/III), the 5-year survival rate was 73%. If the cancer has spread to distant parts of the body (stage IV), the 5-year survival rate was only 17% [54].

In the study population who continued the study, there was a significant difference between the atorvastatin group and the rivaroxaban group in terms of Khorana score (*p*=0.006). In the rivaroxaban group, most patients had Khorana score of 2 (88.5%) and in the atorvastatin group, most patients had Khorana score of 3 (45.8%). Although the number of patients with Khorana score of 3 was higher in the atorvastatin group, the results showed that thrombosis prophylaxis with atorvastatin yielded effective results, and the incidence of DVT was similar between the atorvastatin group and the rivaroxaban group.

The primary efficacy endpoint in this study was the number of DVT case during the 90-day observation period. Only 1 DVT case occurred in the treatment and in the control group, respectively (2.3% vs. 2.2%) (OR 0.953; 95% CI, (0.240-3.971; *p*=1.000). This indicates that the administration of 20 mg atorvastatin daily did not differ significantly from 10 mg rivaroxaban daily for 3 months for thromboprophylaxis of DVT in cancer patients with high risk of thrombosis undergoing chemotherapy.

The incidence of primary efficacy endpoint in this study did not differ from a large-scale study of primary thromboprophylaxis in patients undergoing chemotherapy. The SAVE-ONCO study, a double-blind, multicenter study, compared the ultra-low-molecular-weight heparin semuloparin with placebo to evaluate the effectiveness and safety of VTE prophylaxis in cancer patients undergoing chemotherapy. The results also showed that VTE occurred in only 20 (1.2%) of the 1608 patients receiving semuloparin, compared to 55 (3.4%) of the 1604 patients, which was statistically significant in terms of thromboprophylaxis effectiveness compared to placebo with HR (Hazard Ratio) 0.36; 95% CI, 0.21-0.60; *p*<0.001) [52]. The results of this study seem to indicate that atorvastatin has similar effectiveness to LMWH, but further study is needed to compare the efficacy of atorvastatin with LMWH to prevent DVT.

Likewise, the PROTECHT study, a placebo-controlled, double-blind study, was designed to evaluate the effectiveness of nadroparin for the prophylaxis of venous thromboembolic events in cancer patients undergoing chemotherapy. The results showed that in patients receiving nadroparin, VTE occurred in only 16 (2.1%) of 769 patients, compared to patients receiving placebo where VTE occurred in 15 (3.9%) of 381 patients, which was statistically significant for nadroparin in terms of thromboprophylaxis efficacy compared to placebo (interim-adjusted *p*value = 0.033, relative risk reduction 47.2%, NNT = 53.8) [51].

The low incidence of VTE in the atorvastatin group from this study (2.3%), the SAVE-ONCO (1.2%) and PROTECHT (2.1%) studies is reasonable because the patients received VTE thromboprophylaxis thereby reducing the risk of developing VTE in these patients. The same could explain the underlying mechanism for the low incidence of VTE in the rivaroxaban group (2.2%) of the current study [51, 52].

The results of this study differ from those of the SAVE-ONCO and PROTECHT studies, where the study treatment was found to be statistically more efficacious for preventing VTE events compared to the control. This is due to fact that thromboprophylaxis in the treatment group used an anticoagulant drug, while placebo was used as a comparison. Meanwhile, in this study, the treatment group used atorvastatin while the control group received rivaroxaban, which is an anticoagulant drug that has been used in the guidelines as thromboprophylaxis. The results showed that administering 20 mg atorvastatin daily did not differ significantly from 10 mg rivaroxaban daily for 3 months for the thromboprophylaxis of DVT in cancer patients with high risk of thrombosis undergoing chemotherapy.

In this study, the secondary efficacy endpoint observed in the 90 days observation period were as follows: 1 case of DVT and 16 deaths (17 [40.5%] cases) in the atorvastatin group, and 1 case of DVT and 11 deaths (12 [27.3%] cases) in the rivaroxaban group (OR 1.337; 95% CI, 0.875-2.042; *p* = 0.286).

No significant difference was observed in terms of DVT plus death from any cause in the atorvastatin and rivaroxaban groups. It also showed that that administering 20 mg atorvastatin daily did not differ significantly from 10 mg rivaroxaban daily for 3 months for the thromboprophylaxis of DVT in cancer patients with high risk of thrombosis undergoing chemotherapy.

The primary safety endpoint during the 90 day observation period in this study occurred in 2 (4.8%) and 12 (27.3%) subjects in the atorvastatin and the rivaroxaban group, respectively. There was a significant difference in terms of major bleeding incidence between the atorvastatin group and the rivaroxaban group (OR 0.257; 95% CI, 0.07-0.94; *p* = 0.007). This study observed that the risk of major bleeding was higher in the rivaroxaban group than in the atorvastatin group.

In this study, the secondary safety end point during the 90 day observation period in the atorvastatin group occurred in 2 (4.8%) vs. 6 (13.6%) of patients in the atorvastatin and rivaroxaban group, respectively. There was no significant difference in terms of the incidence of clinically-relevant non-major bleeding between the atorvastatin and rivaroxaban groups (OR 0.32; 95% CI, 0.60-1.67; *p*=0.27). There was a significant difference in the incidence of major bleeding and clinically-relevant non-major bleeding in the atorvastatin group and the rivaroxaban group (*p*=0.001).

Major bleeding mostly occurred in the rivaroxaban group characterized by a clinically significant bleeding with a decrease in hemoglobin levels of 2 g/dL or more requiring transfusion of 2 or more units of blood cells (ISTH criteria) (*n*=7, 31.8%). Clinically relevant non-major bleeding mostly occurred in the rivaroxaban group, with the most frequent location being in the lower gastrointestinal tract (*n*=5, 22.7%).

The results of the current study are in accordance with previous studies, which showed that rivaroxaban had a higher risk of bleeding when administered to patients with gastrointestinal cancer [55, 56]. The most common type of cancer in this study was colorectal cancer in both the atorvastatin group and in the rivaroxaban group (*n*=14 (33.3%) and *n*=17 (38.6%), respectively).

The incidence of primary safety endpoint and secondary safety endpoint in this study differed from previous studies. All three of the SAVE-ONCO, PROTECHT and CASSINI studies used an anticoagulant as a study drug compared to placebo as a control. The results of these 3 studies showed that the incidence of bleeding did not differ significantly between the treatment group and the control group. Meanwhile, the current study showed that both major and minor bleeding occurred more in the control group receiving rivaroxaban compared to the treatment group receiving atorvastatin, and this finding was statistically significant [13, 51, 52].

During the 90-day observation period, the presence of atorvastatin toxicity on liver function and signs of myopathy was evaluated. There were no signs of liver toxicity and myopathy progressing to fatal or nonfatal rhabdomyolysis during the 90-day observation period. There was no significant difference between the atorvastatin and rivaroxaban groups in terms of mean levels of triglycerides and HDL cholesterol during the 90-day observation period. There was a significant difference between total cholesterol and LDL cholesterol levels in the 90-day observation period, with levels in the atorvastatin group being were lower, but remain within normal limits. The results of this study are in accordance with previous studies using atorvastatin. Newman *et al*. analyzed data from 44 studies using Atorvastatin in 16,495 patients. Severe side effects were rare and there were no deaths from treatment with Atorvastatin [33].

Based on the data above, this study showed that administering 20 mg atorvastatin daily did not differ significantly from 10 mg rivaroxaban daily for 3 months for thromboprophylaxis of DVT events in cancer patients with high risk of thrombosis undergoing chemotherapy with a lower risk of bleeding.

The cost-effectiveness comparison analysis between atorvastatin and rivaroxaban showed that atorvastatin was more cost-effective than rivaroxaban as indicated by the ACER (IDR 20,655.00 per effectiveness for atorvastatin vs. IDR 41,453.00 per effectiveness for rivaroxaban).

### Research limitations

In this study nearly 42% of the randomized patients discontinued the study before the 90-day observation period was completed for various reasons. This occurs due to most patients being in advanced cancer stage, leading to unexpected results. The same also occurred in previous studies on DVT prophylaxis to cancer patients, such as the CASSINI [13], PROTECHT [51] and SAVE-ONCO studies [52].

## Conclusion

The administration of 20 mg atorvastatin daily did not differ significantly from 10 mg rivaroxaban daily for 3 months, for the thromboprophylaxis of DVT in cancer patients at high risk of thrombosis undergoing chemotherapy. There were significant differences in the incidence of major bleeding and clinically-relevant non-major bleeding in the atorvastatin group and the rivaroxaban group, with a higher number of bleeding events being in the rivaroxaban group.

The administration of 20 mg atorvastatin daily was less expensive than 10 mg rivaroxaban daily for 3 months, for thromboprophylaxis of DVT events in cancer patients at high risk of thrombosis undergoing chemotherapy.

The presence of limited statistical power and wide confidence intervals in this study prevents us from making a direct conclusion about the possibility of a significant results of this study. It is necessary to conduct a multicenter clinical trial with a larger sample size to further strengthen the evidence of the efficacy of atorvastatin as DVT prophylaxis in cancer patients undergoing chemotherapy, so that atorvastatin could be proposed in clinical guidelines as DVT prophylaxis in cancer patients undergoing chemotherapy.

## Supplementary Information

**Additional file 1: Table S1.** Subjects Discontinued Study. **Table S2.** Rates and Reasons for Early Discontinuation Before Study Completed in the Atorvastatin and Rivaroxaban Groups. **Table S3.** Characteristics of Patients Who Discontinued Study Drug. **Table S4.** Characteristics of Patients Who Remained On Study Drug. **Table S5.** Bleeding Sites in the Atorvastatin and Rivaroxaban Groups. **Figure S1.** Cost Efectiveness diagram.

## Data Availability

Database of all patients and statistical analaysis are available upon request and authorization from Dr. Kariadi Hospital.

## Abbreviations

ACER: Average Cost-Effectiveness Ratio
ASCO: American Society of Clinical Oncology
AST: Aspartate transaminase
ALT: Alanine transaminase
CEA: Cost Effectiveness Analysis
CRP: C-reaktif protein
CI: Confidence Interval
COVID -19: Coronavirus Disease of 2019
CK: Creatinie kinase
CUS: Compression ultrasound
DVT: Deep Vein Thrombosis
ECOG: Eastern Cooperative Oncology Group
5 FU: 5 Fluoro uracil
F1 + 2: Prothrombin fragmen F 1 + 2
FXa: Factor Xa
ICER: Incremental Cost-Effectiveness Ratio/
IL-6: Interleukin 6
ICU: Intensive Care Unit
ISRCTN: The International Standard Randomised Controlled Trial Number
ISTH: International Society on Thrombosis and Hemostasis
ITT: Intention-to-treat
IDR: Indonesian Rupiah
LDL: Low density lipoprotein
LMWH: Low molecular weigh heparin
MAPK: Mitogen-activated protein kinase
NF-κB: Nuclear factor kappa-B
LDL: Low-density lipoprotein
OR: Odds ratio
PAR: Protease-activated receptor
RCT: Randomized controlled trials
TF: Tissue factor
UFH: Unfractionated heparin
VTE: Venous thromboembolism
