# Supplementary material for: The effectiveness of atorvastatin for the prevention of deep vein thrombosis in cancer patients undergoing chemotherapy: A randomised controlled trial: open label
Source: Thromb J. 2023 May 8;21:54. doi: 10.1186/s12959-023-00497-0 (PMC10164452; doi:10.1186/s12959-023-00497-0)
Supplement: Supplementary file 1 — Additional file 1: Table S1. Subjects Discontinued Study. Table S2. Rates and Reasons for Early Discontinuation Before Study Completed in the Atorvastatin and Rivaroxaban Groups. Table S3. Characteristics of Patients Who Discontinued Study Drug. Table S4. Characteristics of Patients Who Remained On Study Drug. Table S5. Bleeding Sites in the Atorvastatin and Rivaroxaban Groups. Figure S1. Cost Efectiveness diagram. [file 12959_2023_497_MOESM1_ESM.docx]

**Table S1. Subjects Discontinued Study.**

| Drop out | Group | | p | OR; 95% CI |
| --- | --- | --- | --- | --- |
|  | Atorvastatin  n=42 | Rivaroxaban  n=44 |  |  |
| Yes | 18 (42,8%) | 18 (40,9%) | 1,000* | 1,042; 0,674-1,611 |
| No | 24 (57,2%) | 26 (59,1%) |  |  |

**Chi Square*

**Table S2. Rates and Reasons for Early Discontinuation Before Study Completed in the Atorvastatin and Rivaroxaban Groups.**

|  | | Atorvastatin  (n=42) | Rivaroxaban  (n=44) | Total  (n=86) | p |
| --- | --- | --- | --- | --- | --- |
| Permanent discontinuation of drugs study | | 18 (42,8%) | 18 (40.9%) |  |  |
| Reasons for permanent discontinuation of drugs study | |  |  |  |  |
|  | Death | 16 (88,9%) | 10 (55,6%) | 26 | 0,043*^⁋^* |
|  | Major Bleeding | 0 (0%) | 4 (22,2%) | 4 |  |
|  | *Primary efficacy end point* | 0 (0%) | 1 (5,6%) | 1 |  |
|  | Patient’s decision | 1 (5,6%) | 1 (5,6%) | 2 |  |
|  | Lost of follow-up | 1 (5,6%) | 0 (0%) | 1 |  |
|  | Researcher’s decision | 0 (0%) | 1 (5,6%) | 1 |  |
|  | Severe Covid | 1 (5,6%) | 0 (0%) | 1 |  |

*^⁋^Mann-Whitney U Test*

**Table S3. Characteristics of Patients Who Discontinued Study Drug**

| Characteristics | | Atorvastatin  (n=18) | Rivaroxaban  (n=18) | Sum  (n=36) | p |
| --- | --- | --- | --- | --- | --- |
| Age (yr), median (min-max) | | 47 (28-60) | 39 (21-55) |  | 0.096^⁋^ |
| Sex, no. (%) | | | | |  |
|  | Male | 11 (61,1%) | 10 (55,6%) | 21 | 1,000* |
|  | Female | 7 (38,9%) | 8 (44,4%) | 15 |  |
| Blood type, no (%) | | | | |  |
|  | O | 7 (38,9%) | 7 (38,9%) | 14 | 1,000* |
|  | Non-O | 11 (61,1%) | 11 (61,1%) | 22 |  |
| Body Mass Index (kg/m^2^), no (%) | | | | |  |
|  | *Underweight* | 7 (38,9%) | 7 (38,9%) | 14 | 0,700^⁋^ |
|  | *Normoweight* | 8 (44,4%) | 10 (55,6%) | 18 |  |
|  | *Overweight/obesity* | 3 (16,7%) | 1 (5,6%) | 4 |  |
| ECOG, no. (%) | | | | |  |
|  | 0 | 7 (38,9%) | 8 (44,4%) | 15 | 0,761^⁋^ |
|  | 1 | 8 (44,4%) | 4 (22,2%) | 12 |  |
|  | 2 | 3 (16,7%) | 6 (33,3%) | 9 |  |
| Khorana score, no. (%) | | | | |  |
|  | Intermediate (2) | 10 (55,6%) | 9 (50%) | 19 | 1,000* |
|  | High (≥3) | 8 (44,4%) | 9 (50%) | 17 |  |
| Primary site of cancer, no. (%) | | | | |  |
|  | **Very high risk of thrombosis** | | | |  |
|  | Pancreas | 3 (16,7%) | 0 (0%) | 3 | 0,003^⁋^ |
|  | Stomach | 2 (11,1%) | 1 (5,6%) | 3 |  |
|  | **High risk of thrombosis** | | | |  |
|  | Lung | 5 (27,8%) | 1 (5,6%) | 6 |  |
|  | Genitourinaries | 1 (5,6%) | 1 (5,6%) | 2 |  |
|  | Gynecology | 0 (0%) | 2 (11,1%) | 2 |  |
|  | Lymphoma | 1 (5,6%) | 3 (16,7%) | 4 |  |
|  | **Average risk of thrombosis** | | | |  |
|  | Colorectal | 4 (22,2%) | 7 (38,9%) | 11 |  |
|  | Breast | 1 (5,6%) | 1 (5,6%) | 2 |  |
|  | Others | 1 (5,6%) | 2 (11,1%) | 3 |  |
| Stage of cancer at diagnosis, no. (%) | | | | |  |
|  | I | 1 (5,6%) | 1 (5,6%) | 2 | 0,043^⁋^ |
|  | II | 3 (16,7%) | 0 (0%) | 3 |  |
|  | III | 6 (33,3%) | 3 (16,7%) | 9 |  |
|  | IV | 8 (44,4%) | 14 (77,8%) | 22 |  |
| Chemotherapy regimen, no. (%) | | | | |  |
|  | 5FU *Based* | 6 (33,3%) | 8 (44,4%) | 14 | 0,639^⁋^ |
|  | Cisplatin *Based* | 8 (44,4%) | 5 (27,8%) | 13 |  |
|  | R-CHOP | 0 (0%) | 3 (16,7%) | 3 |  |
|  | BEP | 1 (5,6%) | 0 (0%) | 1 |  |
|  | Taxane *Monotherapy* | 1 (5,6%) | 1 (5,6%) | 2 |  |
|  | Anthracycline *Based* | 1 (5,6%) | 1 (5,6%) | 2 |  |
|  | GRALL-LYSA | 1 (5,6%) | 0 (0%) | 1 |  |
| Laboratory parameters | | | | |  |
|  | Hemoglobin (g/dL) | 11,1 (8,5-14,9) | 10,9 (7,3-12,9) |  | 0,217^⁋^ |
|  | Leukocyte (x10^3^/uL) | 15 (49-37) | 13,5 (5,9-21,8) |  | 0,613^⁋^ |
|  | Platelet (x10^3^/uL) | 470(194-612) | 480 rb (25-951) |  | 0,448^⁋^ |
| **Chi Square; ^⁋^Mann-Whitney U Test* | | | | | |

**Table S4. Characteristics of Patients Who Remained On Study Drug**

| Characteristics | | Atorvastatin  (n=24) | Rivaroxaban  (n=26) | Total  (n=50) | p |
| --- | --- | --- | --- | --- | --- |
| Age (yr), median (min – max) | | 43,5 (19-60) | 42 (20-60) |  | 0,726^⁋^ |
| Sex, no. (%) | | | | |  |
|  | Male | 10 (41,7%) | 14 (53,8%) | 24 | 0,388* |
|  | Female | 14 (58,3%) | 12 (46,2%) | 26 |  |
| Blood type, no. (%) | | | | |  |
|  | O | 12 (50%) | 10 (38,5%) | 22 | 0,411* |
|  | Non-O | 12 (50%) | 16 (61,3%) | 28 |  |
| Body Mass Index (kg/m^2^), no. (%) | | | | |  |
|  | *Underweight* | 7 (29,2%) | 13 (50%) | 20 | 0,075^⁋^ |
|  | *Normoweight* | 13 (54,2%) | 12 (46,2%) | 26 |  |
|  | *Overweight/obesity* | 4 (16,7%) | 1 (3,8%) | 6 |  |
| ECOG, no (%) | | | | |  |
|  | 0 | 19 (79,2%) | 21 (80,8%) | 41 | 0,747^⁋^ |
|  | 1 | 4 (16,7%) | 4 (15,4%) | 9 |  |
|  | 2 | 1 (4,2%) | 1 (3,8%) | 2 |  |
| Khorana score, no. (%) | | | | |  |
|  | Intermediate (2) | 13 (54,2%) | 23 (88,5%) | 36 | 0,006* |
|  | High (≥3) | 11 (45,8%) | 3 (11,5%) | 14 |  |
| Primary site of cancer, no (%) | | | | |  |
|  | **Very high risk of thrombosis** | | | |  |
|  | Pancreas | 2 (8,3%) | 2 (7,7%) | 4 | 0,493^⁋^ |
|  | **High risk of thrombosis** | | | |  |
|  | Lung | 3 (12,5%) | 6 (23,1%) | 9 |  |
|  | Genitourinaries | 2 (8,3%) | 1 (3,8%) | 3 |  |
|  | Lymphoma | 3 (12,5%) | 2 (7,7%) | 5 |  |
|  | **Average risk of thrombosis** | | | |  |
|  | Colorectal | 10 (41,7%) | 10 (38,5%) | 20 |  |
|  | Breast | 1 (4,2%) | 1 (3,8%) | 2 |  |
|  | Sarkoma | 0 (0%) | 2 (7,7%) | 2 |  |
|  | Others | 3 (12,5%) | 2 (7,7%) | 5 |  |
| Stage of cancer at diagnosis, no (%) | | | | |  |
|  | I | 1 (4,2%) | 3 (11,5%) | 4 | 0,1^⁋^ |
|  | II | 2 (8,3%) | 5 (19,2%) | 7 |  |
|  | III | 7 (29,2%) | 8 (30,8%) | 15 |  |
|  | IV | 14 (58,3%) | 10 (38,5%) | 24 |  |
| Chemotherapy regimen, no. (%) | | | | |  |
|  | 5FU *Based* | 14 (58,3%) | 12 (46,2%) | 26 | 0,489^⁋^ |
|  | Cisplatin *Based* | 6 (25%) | 9 (34,6%) | 15 |  |
|  | R-CHOP | 1 (4,2%) | 1 (3,8%) | 2 |  |
|  | BEP | 0 (0%) | 1 (3,8%) | 1 |  |
|  | Taxane *Monotherapy* | 1 (4,2%) | 1 (3,8%) | 2 |  |
|  | Anthracycline *Based* | 0 (0%) | 1 (3,8%) | 1 |  |
|  | ABVD | 1 (4,2%) | 1 (3,8%) | 2 |  |
|  | De Angelis | 1 (4,2%) | 0 (0%) | 1 |  |
| Laboratory Parameters | | | | |  |
|  | Hemoglobin (g/dL) | 11,05 (7,7-15,3) | 11,85 (7.9-15,9) |  | 0,122^⁋^ |
|  | Lekosit (x10^3^/uL) | 12,3 (6,6-21,6) | 11,3 (5.1-20,4) |  | 0,203^⁋^ |
|  | Trombosit (x10^3^/uL) | 439 (357-707) | 429 (238-714) |  | 0,627^⁋^ |
| **Chi Square;* *^⁋^Mann Whitney U Test* | | | | | |

**Table S5. Bleeding Sites in the Atorvastatin and Rivaroxaban Groups**

| Location | Atorvastatin | | Rivaroxaban | | Total |
| --- | --- | --- | --- | --- | --- |
|  | Major Bleeding | Clinically relevant non-major bleeding | Major Bleeding | Clinically relevant non-major bleeding |  |
| Lower GI Tract | 0 (0%) | 1 (4,5%) | 1 (4,5%) | 5 (22,7%) | 7 (31,8%) |
| Upper GI Tract | 0 (0%) | 0 (0%) | 1 (4,5%) | 0 (0%) | 1 (4,5%) |
| Breast | 0 (0%) | 1 (4,5%) | 1 (4,5%) | 0 (0%) | 2 (9%) |
| Gynecology Organ | 0 (0%) | 0 (0%) | 2 (9%) | 0 (0%) | 2 (9%) |
| Lower Extremities | 0 (0%) | 0 (0%) | 0 (0%) | 1 (4,5%) | 1 (4,5) |
| Decrease in Hb > 2 g/dL | 2 (9%) | 0(0%) | 7 (31,8%) | 0(0%) | 9 (40,9%) |
| Total | 2 (9%) | 2 (9%) | 12 (54,6%) | 6 (27,2%) | 22 (100%) |

**Figure S1. Cost Efectiveness diagram.** The third stage of this analysis is the positioning of alternative thrombosis prophylaxis based on the cost-effectiveness diagram.^1^ The positioning of alternative treatments is seen from the average total cost of therapy and effectiveness. The desired alternative for thrombosis prophylaxis in cancer patients in this study is atorvastatin. The position of atorvastatin is in column D, which means that atorvastatin has similar effectiveness and lower cost compared to rivaroxaban, so there is no need to calculate Incremental Cost-Effectiveness Ratio (ICER).

| **Effectiveness - Cost** | **Lower cost** | **Same cost** | **Higher Cost** |
| --- | --- | --- | --- |
| Lower effectiveness | A  (Need ICER calculation) | B | C  (Dominan) |
| Same effectiveness | D | E | F |
| Higher effectiveness | G  (Dominan) | H | I  (Need ICER calculation) |

**REFERENCE**

1. Sarnianto P, Fadia Z, Gusnellyanti E, editor. Pedoman Penerapan Kajian Farmakoekonomi [Internet]. Kemenkes RI; 2013. Available from: http://farmalkes.kemkes.go.id
